# UNDERSTANDING THE ROLE OF CULTURE IN SHAPING ATTITUDES AND BELIEFS ON URINARY INCONTINENCE: A SCOPING REVIEW

**DOI:** 10.1093/geroni/igae098.3761

**Published:** 2024-12-31

**Authors:** Simran Panesar, Saima Rajabali, Adrian Wagg

**Affiliations:** University of Alberta, Edmonton, Alberta, Canada; University of Alberta, Edmonton, Alberta, Canada; University of Alberta, Edmonton, Alberta, Canada

## Abstract

Urinary incontinence (UI) is a common condition among older adults with diverse consequences for health and well-being. Shame, stigma, and cultural perspectives can prevent treatment-seeking behaviour. There is a scarcity of literature that explores the role that culture, beyond stigma, plays in shaping attitudes to, beliefs about, and management practices of UI. This review investigates and describes what is known about the role of culture in shaping the attitudes and beliefs on UI to identify gaps in the literature and direct future research. Following the Joanna Briggs Institute (JBI) method for scoping reviews, the MEDLINE, EMBASE, PsychINFO, CINAHL, EBSCOhost, and SCOPUS databases were searched, without any restriction on language and publication date. A full extraction was conducted of seventeen articles that met the inclusion criteria. Key themes revealed were the stress of managing UI and devout cleanliness for religious activities among Muslim women, a norm of secrecy/silence around UI for Latina women, the potent role of caste discrimination on pelvic health-seeking behaviours in parts of India, and the intersection of UI with cultural belief systems like Confucianism, which prioritize maintaining respectability, challenged by the taboo condition of UI. Although several studies emphasized the surface-level stigma of UI, there is a dearth of literature exploring culture and UI. Further research is needed to inform health promotion efforts regarding UI, which consider the severe emotional toll of UI stemming from the isolation it can create, segregating those that experience it from full participation in society.

